# A Modified Chinese Herbal Decoction (Kai-Xin-San) Promotes NGF-Induced Neuronal Differentiation in PC12 Cells via Up-Regulating Trk A Signaling

**DOI:** 10.3389/fcell.2017.00118

**Published:** 2017-12-22

**Authors:** Lu Yan, Min Wei, Amy G. Gong, Pingping Song, Jianshu Lou, Cathy W. Bi, Sherry L. Xu, Aizhen Xiong, Tina T. Dong, Karl W. Tsim

**Affiliations:** ^1^Shenzhen Research Institute, Hong Kong University of Science and Technology, Shenzhez, China; ^2^Institute of Botany, Jiangsu Province and Chinese Academy of Sciences, Nanjing Botanical Garden Mem. Sun Yat-Sen, Nanjing, China; ^3^Jiangsu Key Laboratory for the Research and Utilization of Plant Resources, Nanjing, China; ^4^Division of Life Science and Center for Chinese Medicine, Hong Kong University of Science and Technology, Hong Kong, China

**Keywords:** Kai-Xin-San, depression, paired-herbs, neuronal differentiation, Trk A

## Abstract

Kai-Xin-San (KXS), a Chinese herbal decoction, has been applied to medical care of depression for thousands of years. It is composed of two functional paired-herbs: Ginseng Radix et Rhizoma (GR)-Polygalae Radix (PR) and Acori Tatarinowii Rhizoma (ATR)-Poria (PO). The compatibility of the paired-herbs has been frequently changed to meet the criteria of syndrome differentiation and treatment variation. Currently, a modified KXS (namely KXS_2012_) was prepared by optimizing the combinations of GR-PR and ATR-PO: the new herbal formula was shown to be very effective in animal studies. However, the cellular mechanism of KXS_2012_ against depression has not been fully investigated. Here, the study on KXS_2012_-induced neuronal differentiation in cultured PC12 cells was analyzed. In PC12 cultures, single application of KXS_2012_ showed no effect on the neuronal differentiation, but which showed robust effects in potentiating nerve growth factor (NGF)-induced neurite outgrowth and neurofilament expression. The potentiating effect of KXS_2012_ was mediated through NGF receptor, tropomyosin receptor kinase (Trk) A: because the receptor expression and activity was markedly up-regulated in the presence of KXS_2012_, and the potentiating effect was blocked by k252a, an inhibitor of Trk A. Our current results in cell cultures fully support the therapeutic efficacy of KXS_2012_ against depression.

## Introduction

Depression, namely major depressive disorder, is a serious mental illness characterized by constant feeling of low mood, low self-esteem, and loss of interest or pleasure (Barlow and Durand, 2011; Ferrari and Villa, 2016). The major pathological alteration of depression includes neuron reduction, disorder in neurotransmitter system and loss of neurotrophic factors; these changes are more robust in hippocampus and cortex (Krishnan and Nestler, 2008; Masi and Brovedani, 2011). The dysfunction in neurotransmitter system has been widely recognized and considered as one of the most severe pathogenesis. However, anti-depressants targeted on regulating neurotransmitter levels are not able to exert an effect to all the patients, indicating the complex pathogenesis during depression (Pehrson and Sanchez, 2014; Duman and Aghajanian, 2015). In the brain of depressed patients, nerve reduction resulted from retarded neurogenesis deposits that depression is largely caused by an impairment of the brain's ability to maintain neurogenesis. The disorder is reversible when neurogenesis is improved. During neurogenesis, neuronal differentiation having the neurite outgrowth is of great importance for brain function and low mood regulation. Thus, the induction of neuronal differentiation predicts a novel and effective therapeutic target for development of anti-depressants (Qin et al., 2008).

Kai-Xin-San (KXS) from *Beiji Qianjin Yaofang* <*Thousand Formulae for Emergency*> by Sun Simiao (581–685 A.D.) in China has been applied to medical care of depression for thousands of years (Sun, 1997; Yasunori, 2011). KXS composes two functional paired-herbs, i.e., Ginseng Radix et Rhizoma (GR; root and rhizome of *Panax ginseng* C. A. Mey.; Araliaceae family)—Polygalae Radix (PR; root of *Polygala tenuifolia* Wild.; Polygalaceae family) and Acori Tatarinowii Rhizoma (ATR; rhizome of *Acorus tatarinowii* Schott; Acoraceae family)—Poria [PO; sclerotium of *Poria cocos* (Schw.) Wolf; Polyporaceae family]. The make-up of the paired-herbs contributes to anti-depressant functions of KXS. The administration of KXS alleviated depressive symptoms in animal studies with the mechanism of restoring the levels of neurotransmitters and neurotrophic factors in the brain (Dang et al., 2009; Zhu et al., 2012), as well as in cultured astrocytes and neurons (Zhu et al., 2013; Hu et al., 2014). However, the combination of GR-PR and ATR-PO has been frequently changed to meet the criteria of syndrome differentiation and treatment variation, which hinders the development of anti-depressant. Thus, KXS_2012_ (GR-PR: ATR-PO = 1:5) was prepared by optimizing the compatibility of GR-PR and ATR-PO (Yan et al., 2015). This new formulation was able to significantly alleviate depression-like symptoms and to regulate neurotransmitter and neurotrophic factor levels in animal study (Yan et al., 2016). However, the cellular mechanism of KXS_2012_ against depression has not been fully illustrated.

Here, we evaluated the function of KXS_2012_ against depression via promoting neuronal differentiation, an important stage of neurogenesis. Among different cell models for neuronal differentiation, cultured PC12 cell is usually selected for detection of neuronal differentiation in responding to various stimuli, e.g., NGF (Lortie et al., 2005; Marín-Vicente et al., 2011). The status of differentiated PC12 cells was determined morphological through measuring neurite outgrowth and biochemically through analyzing the expression of neurofilaments, i.e., neurofilament is the major structural components of differentiated neurons (Schimmelpfeng et al., 2004; Xu et al., 2012). In neurons, NGF activates its receptor, Trk A, to promote neuronal differentiation. Thus, the Trk A signaling was studied to predict the potential cellular mechanism against depression. Our results could accelerate the development of new therapy for anti-depression.

## Materials and methods

### Preparation of herbal decoction

The plant materials, purchased from Qinping Market in Guangzhou China, were morphologically authenticated by one of the authors, Dr. Tina T. Dong. The corresponding voucher specimens were deposited in Center for Chinese Medicine of The Hong Kong University of Science and Technology. The herbs were tested to be qualified according to the requirements of Chinese Pharmacopeia (2015 Edition) and Hong Kong Materia Medica Standards.

According to *Yi Xin Fang* (Yasunori, 2011), GR-PR and ATR-PO were prepared according to the weight ratio of 1:1 and 1:2, respectively. In preparing the herbal extracts, 20 g of herb mixture was boiled in 160 mL of water for 2 h and extracted twice. The extracts were combined, dried under vacuum and stored at −80°C. KXS_2012_ was obtained by mixing the water extracts of GR-PR and ATR-PO together in 1:5 weight ratio. The quality control of herbal extracts was described in previous study (Yan et al., 2015). The extracts were solubilized in dimethylsulfoxide (DMSO) to give stock solution at a series of concentration from 15 to 50 mg/mL and stored at −20°C.

### Cell culture

PC12 cells, originated from rat adrenal medulla, were purchased from American Type Culture Collection (ATCC, Manassas, VA). Cells were cultured in Dulbecco's modified Eagle's medium (DMEM), supplemented with 6% fetal bovine serum (FBS) and horse serum (HS), 100 units/mL penicillin and 100 μg/mL streptomycin in a humidified CO_2_ (7.5%) incubator at 37°C. Fresh medium was applied every other day. All culture reagents were purchased from Life Technologies (Grand Island, NY).

### Herbal treatment

Cultured PC12 cells, after serum starvation for 3 h in DMEM having 1% FBS, HS, penicillin, and streptomycin, were treated with KXS_2012_ for 48 h for western blotting, or 24 h for real-time quantitative PCR. The cell viability assay was performed to determine a safe concentration range (0–100 μg/mL) of each extract, at which all extracts did not induce cell proliferation or death (Yan et al., 2015). In the co-treatment, cultured PC12 cells were treated with NGF (1.5 ng/mL) and KXS_2012_ (15 μg/mL) for 24 h for western blotting assay. In the pre-treatment, PC12 cells were treated with herbal extract for 48 h and washed by 1x PBS twice before NGF (1.5 ng/mL) application for 24 h. In phosphorylation study, the cultures were starved for 5 h in DMEM before NGF (1.5 ng/mL) or herbal application.

### Polymerase chain reaction

Total RNA was isolated from cell cultures by RNAzol RT reagent (Molecular Research Center, Cincinnati, OH) according to the manufacture's instruction. The amounts of RNAs were detected by UV absorbance at 260 nm. The total RNA was used to do the reverse transcription with moloney murine leukemia virus (MMLV) reverse transcriptase (Life Technologies), according to the protocol provided by the manufacturer. Real-time PCR was performed by using FastStart SYBR Green Master (Roche, Indianapolis, IN), according to the manufacturer's instruction. The SYBR green signal was detected by Mx3000P^TM^ muitiplex quantitative PCR machine (BD Biosciences Clontech, San Jose, CA).

### SDS-page and immunoblotting

After the treatment, the cells were solubilized in lysis buffer containing 0.125 M Tris-HCl, PH6.8, 4% SDS, 20% glycerol, 2% 2-mercaptoethanol, and analyzed immediately or stored frozen at −20°C. Proteins were separated on 8% SDS-polyacrylamide gels and transferred to a nitrocellulose. Transfer and equal loading of the samples was confirmed by staining the ponceau-S. The nitrocellulose was blocked with 5% fat-free milk in Tris-buffer saline/0.1% Tween 20 (TBS-T), and then incubated in the primary antibodies diluted in 2.5% fat-free milk in TBS-T over night at 4°C. The primary antibodies were: anti-NF200 (Sigma-Aldrich, St. Louis, MO, N4142, 1:1,000), anti-NF160 (Sigma-Aldrich, N2787, 1:5,000), anti-NF68 (Sigma-Aldrich, N5139, 1:2,000), anti-GAPDH (Abcam Ltd, Cambridge, UK, ab9485, 1:1,000,000), anti-phospho-Trk A (Cell Signaling, Danvers, MA, 9141, 1:1,000), anti-Trk A (Cell Signaling, 2505, 1:1,000), anti-phospho-Erk1/2 (Cell Signaling, 9101, 1:1,000), anti-Erk1/2 (Abcam, ab17942, 1:5,000), anti-phospho-CREB (Cell Signaling, 9198, 1:1,000), and anti-CREB (Cell Signaling, 9197, 1:5,000). After that, the nitrocellulose was rinsed with TBS-T and incubated for 2 h at room temperature in peroxidase (HRP)-conjugated anti-mouse secondary antibody, or peroxidase (HRP)-conjugated anti-rabbit secondary antibody (Life Technologies, 31430 or 31460, 1:5,000), diluted with 2.5% fat-free milk in TBS-T. After intensive washing with TBS-T, the immune complexes were visualized using the enhanced chemiluminescence (ECL) method (GE Healthcare, Piscataway, NJ). The intensities of bands in control and samples, run on the same gel and under strictly standardized ECL conditions, were compared on an image analyzer, using a calibration plot constructed from a parallel gel with serial dilutions of one of the sample.

### Neurite outgrowth assay

A light microscope (Diagnostic Instruments, Sterling Heights, MI) equipped with a phase-contrast condenser, 10x objective lens and a digital camera (Diagnostic Instruments) was used to capture the images with the manual setting. For analyzing the number and length of neurite, ~100 cells were counted from at least 10 randomly chosen visual fields for each culture. Using the photoshop software, the cells were then analyzed for the number and length of neurite. The cells were scored as differentiated if one or more neuritis was longer than diameter of the cell body, and they were classified to different groups according to the length of neurite that it possessed, i.e., < 15, 15–30, and >30 μm (Xu et al., 2012). The number of neurite per cell was counting only those neurite that was longer than cell body.

### Statistical analysis

All data were analyzed using one-way ANOVA followed by the Students *t*-test. Statistical significance were classed as ^*^P < 0.05; ^**^P < 0.01; ^***^P < 0.001.

## Results

### Standardization of herbal extracts

**Figure 1:**
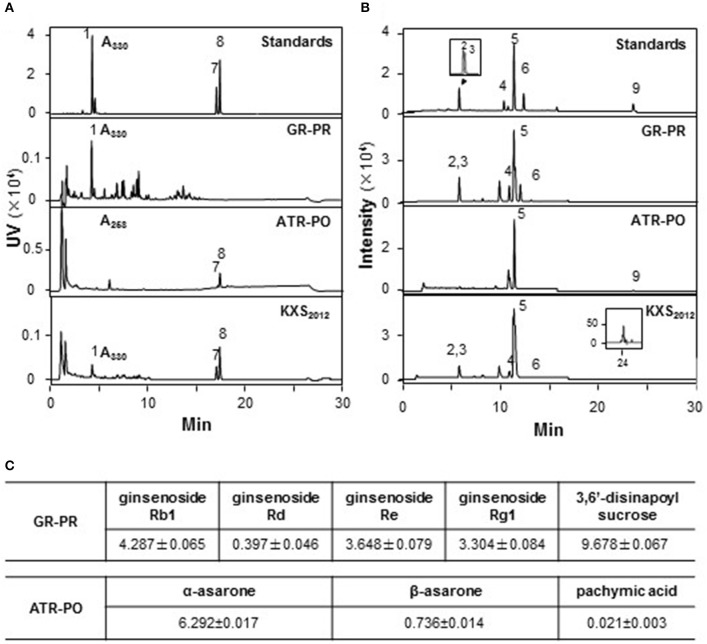
Standardization of herbal extracts. **(A)** The standardization of herbal extracts was described in our previous report (Zhu et al., 2010). The identification of 3, 6′-disnapoyl sucrose (330 nm) (1), α-asarone (258 nm) (8), and β-asarone (258 nm) (7) was made by a HPLC coupled with a DAD detector in paired-herb extracts. This HPLC profile served as fingerprint and determination of marker chemicals. **(B)** In chemical assessment, the identification of ginsenoside Rg_1_ (2), Re (3), Rb_1_ (4), Rd (6), astragaloside IV (5) and pachymic acid (9) was made by a MS detector in standard markers and paired-herb extracts. The amounts of each chemical in mg/g of dried herbal extract were shown in **(C)**, Mean ± SEM, *n* = 3.

According to ancient preparation method of herbal mixture, GR-PR and ATR-PO were prepared in a weight ratio of 1:1 and 1:2, respectively. The extraction efficiency of GR-PR and ATR-PO were about 25.79 ± 3.25 and 10.32 ± 2.78%, respectively (Mean ± *SD, n* = 3). Two approaches were selected for the quality control of paired-herb extracts: (i) chemical fingerprinting; and (ii) minimal-marker requirement. The identified chemicals were shown in fingerprints (Figures 1A,B), according to our published reports (Zhu et al., 2010; Yan et al., 2015). Eight chemical markers, i.e., GR-derived ginsenosides (Rb_1_, Rd, Re, Rg_1_), PR-derived 3,6′-disinapoyl sucrose (330 nm), ATR-derived α-asarone (258 nm), and β-asarone (258 nm) and PO-derived pachymic acid, were selected for chemical quantification (Figure 1). In GR-PR extract, the amounts of Rb_1_, Rd, Re, Rg_1_ and 3,6′-disinapoyl sucrose were about 4.287 ± 0.065, 0.397 ± 0.046, 3.648 ± 0.079, 3.304 ± 0.084, and 9.678 ± 0.067, respectively, in mg/g of dried herbal extract. In ATR-PO extract, the amounts of α-asarone, β-asarone, and pachymic acid were 6.292 ± 0.017, 0.736 ± 0.014, and 0.021 ± 0.003 in mg/g of dried herbal extract (Mean ± *SD, n* = 3) (Figure 1C). The established chemical parameters served as the control for repeatability of below biochemical analyses. Thus, KXS_2012_ was referring to a mixture of GR-PR extract plus ATR-PO extract at 1:5 weight ratio.

### KXS_2012_ promotes NGF-induced neuronal differentiation

**Figure 2:**
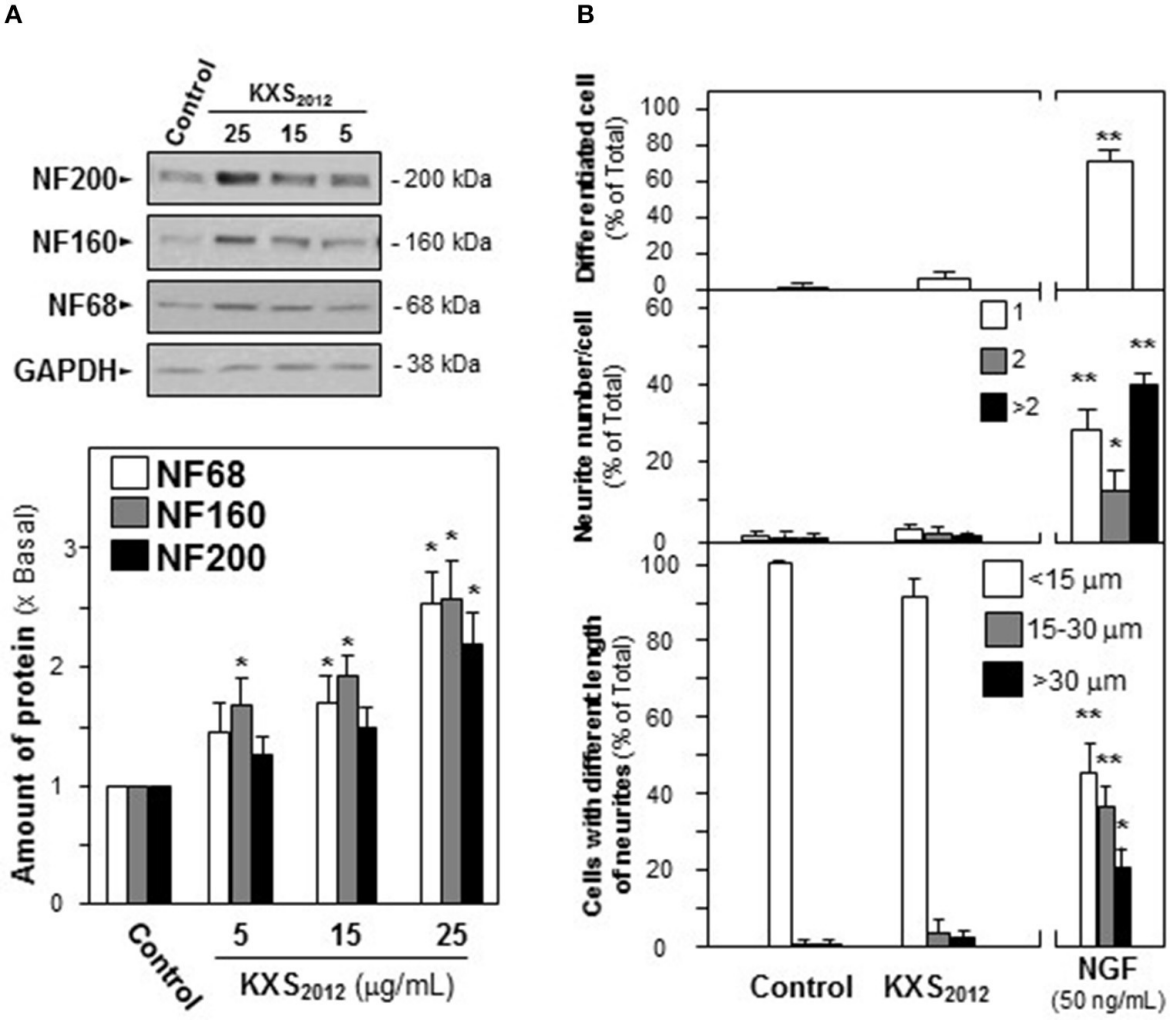
KXS_2012_ does not induce neuronal differentiation. **(A)** KXS_2012_ (5–25 μg/mL) were applied onto cultured PC12 cells for 48 h. The expressions of neurofilaments (NF68, NF160, and NF200) were determined by specific antibodies. Glyceraldehyde-3-phosphate dehydrogenase (GAPDH) served as a loading control. Quantification plot was shown in histograms. **(B)** Cultured PC12 cells were treated as in **(A)**. The cells were fixed with ice-cold 4% paraformaldehyde. The % of differentiated cells (upper panel), the number of neurite per cell (middle panel), and the length of neurite (lower panel) were counted as described in the Materials and Methods section. Bar = 10 μm. NGF (50 ng/mL) served as a positive control. Representative images were shown. Values are expressed as % of total cells in 100 counted cells, Mean ± SEM, *n* = 4. ^*^p < 0.05 and ^**^p < 0.01 compared to the control.

The cellular study of KXS_2012_ on PC12 cells was employed to investigate the function of herbal extracts in neuronal differentiation. The neuronal differentiation of PC12 cells could be determined morphologically in measuring the length of neurite: NGF induced the neurite outgrowth in a dose-dependent manner (Supplementary Figure 1). Three mammalian neurofilament subunits, NF68 (~68 kDa), NF160 (~160 kDa), and NF200 (~200 kDa), form hetero-dimers in making the structural domain of neurite (Schimmelpfeng et al., 2004; Xiong et al., 2016). Application of NGF in cultured PC12 cells also induced the expressions of neurofilaments in dose-dependent manners (Supplementary Figure 2). In cultured PC 12 cells, application of KXS_2012_ caused up-regulation of neurofilaments, NF68, NF160, and NF200, in dose-dependent manners (Figure 2A). The treatment of KXS_2012_ at 25 μg/mL slightly induced neurite outgrowth in PC12 cells. As compared to the control group, the effect possessed no significant difference neither on the length of neurite nor the number of neurite (Figure 2B). Thus, the herbal extract was not able to promote neuronal differentiation as single treatment.

**Figure 3:**
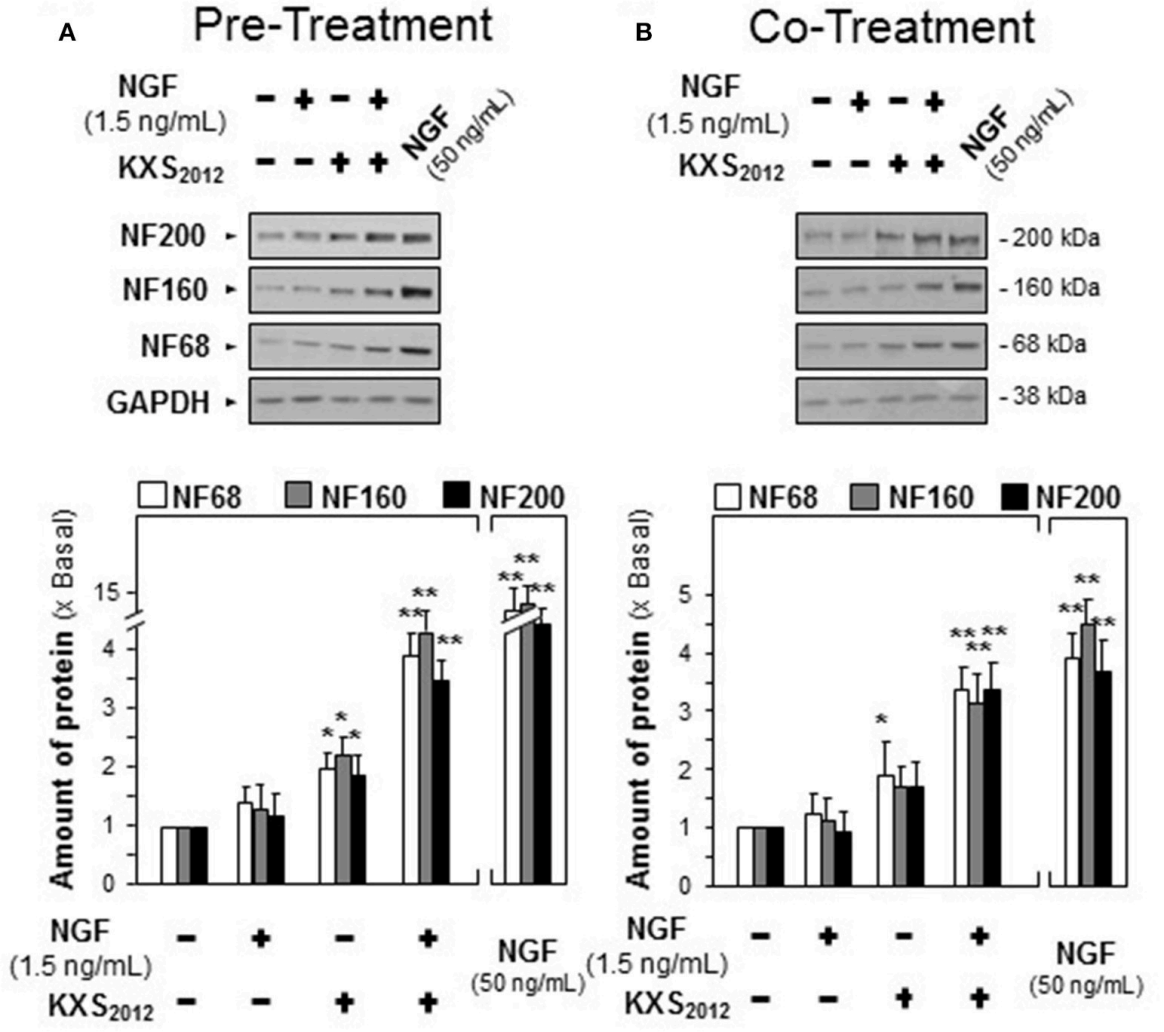
KXS_2012_ promotes NGF-induced neurofilament expression. **(A)** KXS_2012_ (15 μg/mL) was applied onto cultured PC12 cells for 48 h before application of NGF (1.5 ng/mL) for 24 h. **(B)** KXS_2012_ (15 μg/mL) and NGF (1.5 ng/mL) were co-applied onto cultured PC12 cells for 24 h. NGF (50 ng/mL) was applied as a positive control. The cell lysates were collected to determine the amounts of NF68, NF160, and NF200. GADPH served as a loading control. Values are expressed as × Basal where control value is set as 1, Mean ± SEM, *n* = 4. ^*^p<0.05 and ^**^p<0.01 compared to the control.

**Figure 4:**
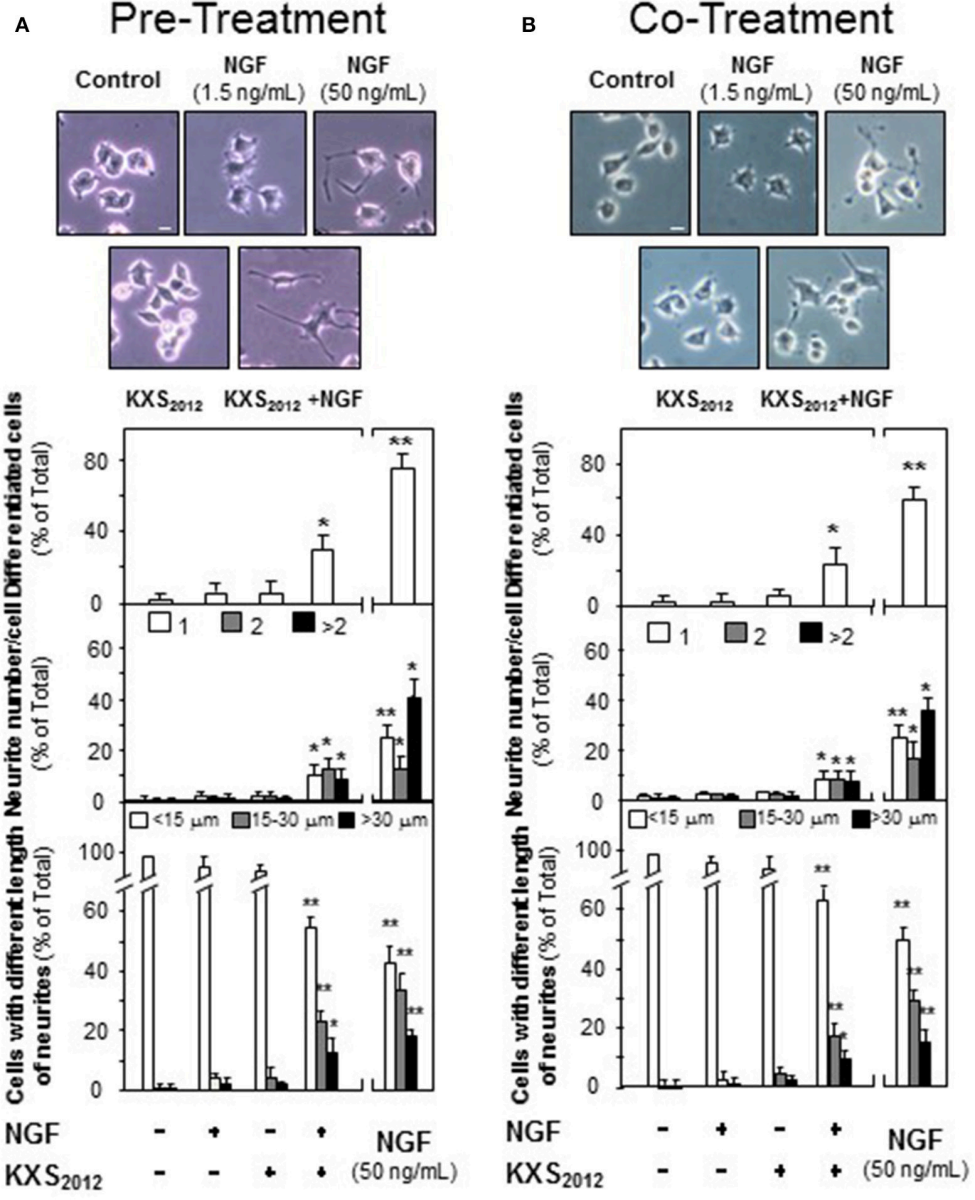
KXS_2012_ promotes NGF-induced neurite outgrowth. **(A)** KXS_2012_ (15 μg/mL) was applied onto cultured PC12 cells for 48 h before NGF application (1.5 ng/mL) for 24 h. **(B)** KXS_2012_ (15 μg/mL) and NGF (1.5 ng/mL) were applied onto cultured PC12 cells for 24 h. NGF (50 ng/mL) was applied as a positive control. Cells were fixed with ice-cold 4% paraformaldehyde, and the extension of neurite was revealed. Bar = 10 μm. The % of differentiated cells (upper panel), the number of neurite per cell (middle panel), and the length of neurite (lower panel) were counted as described in the Materials and Methods section. Values are expressed as % of total cells in 100 counted cells, Mean ± SEM, *n* = 4. ^*^p < 0.05 and ^**^p < 0.01 compared to the control.

In depressed mammalian brain, the level of NGF is reduced, and NGF-induced neuronal differentiation is down-regulated. Thus, the effect of KXS_2012_ in the presence of low level of NGF in neuronal differentiation was determined here. KXS_2012_ at 15 μg/mL, a concentration just barely showed induction of neurofilament expressions, was applied together with a small amount of NGF onto cultured PC12 cells by the methods of pre- and co-treatments. A suitable concentration of NGF was selected: this concentration should have no or little effect on induction of neurite outgrowth or neurofilament expressions. NGF at 1.5 ng/mL was selected in pre-treatment and co-treatment studies (see Supplementary Figures 1, 2): this concentration was to ensure the responses from a low amount of KXS_2012_ could be revealed. In both pre- and co-treatments, the inductions of NF68, NF160, and NF200, induced by KXS_2012_ plus NGF, were significantly higher at 3- to 4-folds (Figure 3). However, the effect triggered by pre-treatment was more robust than that of co-treatment. In neurite outgrowth assay, the number of differentiated cells by pre-treatment of KXS_2012_ and NGF was reached to ~30% (Figure 4A), and ~20% increase was revealed in the co-treatment (Figure 4B). By counting the number of neurite per cell, the pre- and co-treatment of KXS_2012_ plus low dose of NGF caused an increase, significantly, by about 10% of cells having neurite (Figure 4). In addition, the differentiated cells, induced by KXS_2012_ plus low amount of NGF, contained longer neurite, e.g., >15 and >30 μm (Figure 4). Compared to pre-treatment study, the inductive role of KXS_2012_ showed less robust effect in promoting NGF-induced neurite outgrowth by co-treatment (Figure 4).

### The effect of KXS_2012_ on neuronal differentiation via up-regulating trk a signaling

**Figure 5:**
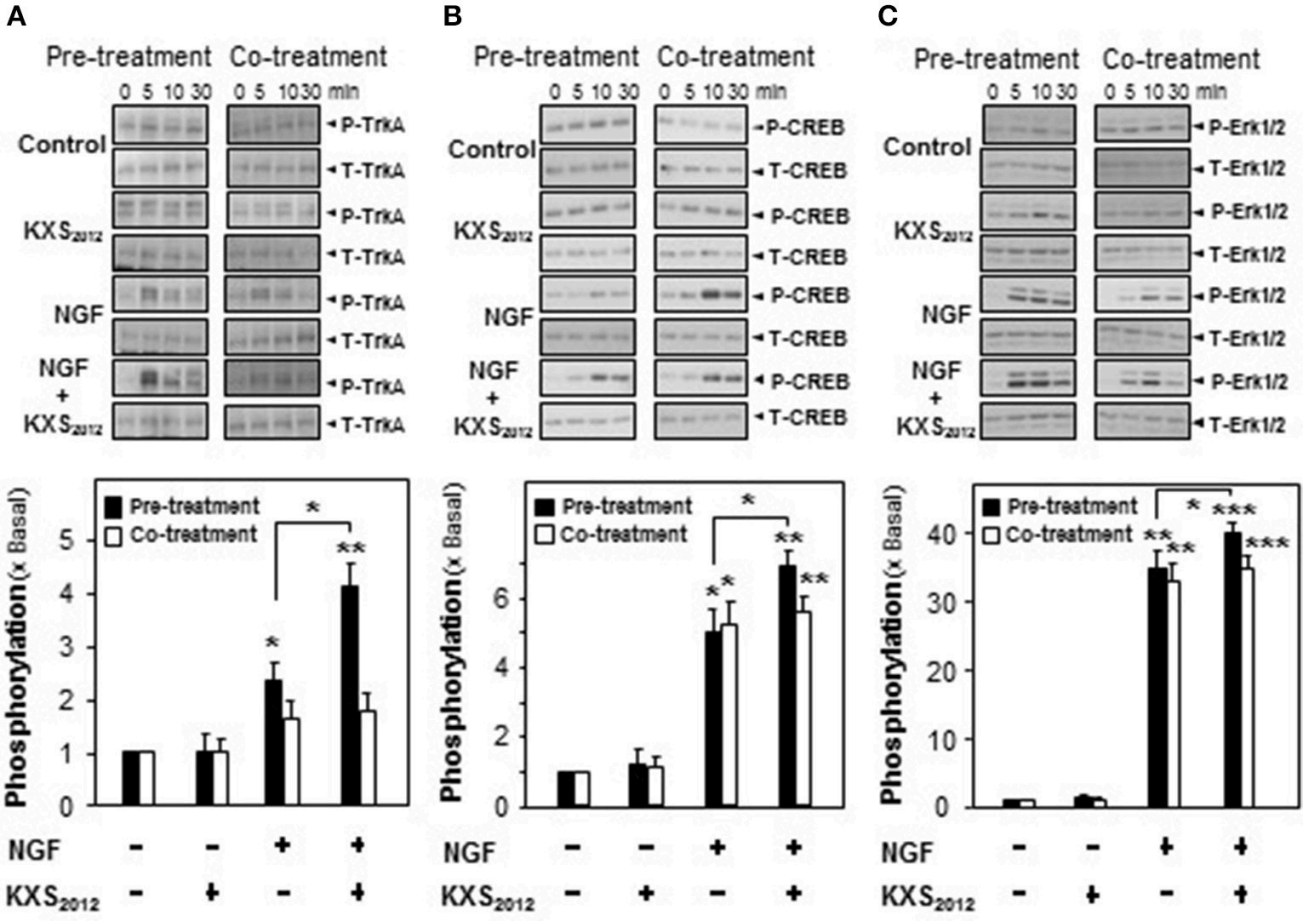
KXS_2012_ promotes NGF-induced phosphorylation. In pre-treatment, KXS_2012_ (15 μg/mL) was applied onto cultured PC12 cells for 48 h before serum starvation for 5 h, with or without application of NGF (1.5 ng/mL), for different time. In co-treatment, cultured PC12 cells, serum starvation for 5 h, were co-treated with NGF (1.5 ng/mL), KXS_2012_ (15 μg/mL) and KXS_2012_ (15 μg/mL) + NGF (1.5 ng/mL) for different time. **(A)** Total Trk A and phosphorylated Trk A were revealed by using specific antibodies. **(B)** Total CREB and phosphorylated CREB were revealed by using specific antibodies. **(C)** Total Erk1/2 and phosphorylated Erk1/2 were revealed by using specific antibodies. Quantification plot of the phosphorylation level in treatment of 5 **(A)** and 10 **(B,C)** min was shown. Values are expressed × Basal, where control value is set as 1, Mean ± SEM, *n* = 4. ^*^p < 0.05, ^**^p < 0.01, and ^***^p < 0.001 compared to the control.

NGF achieves its function by binding to and activating Trk A in PC12 cells. The NGF-activated Trk A stimulates downstream signaling pathways leading to neuronal differentiation and promoting cell survival (Zhang et al., 2000; Vaudry et al., 2002). The role of KXS_2012_ in phosphorylating Trk A was tested here. NGF induced Trk A phosphorylation in a dose-dependent manner, and NGF at 1.5 ng/mL was selected in phosphorylation study, which barely showed phosphorylation capability (Supplementary Figure 3). KXS_2012_ or co-treatment procedure did not effectively phosphorylate Trk A; however, the pre-treatment of KXS_2012_ together with low level of NGF showed much better responsiveness (Figure 5A).

In addition to Trk A, the downstream effectors of CREB and Erk1/2 were also determined here by pre- and co-treatment of KXS_2012_ with low amount of NGF. In cultured PC12 cells, NGF induced CREB phosphorylation (~40 kDa) at ~5-folds; however, co-treatment with KXS_2012_ did not show significant difference, as compared to NGF single treatment (Figure 5B). In contrast, the phosphorylation of CREB was significantly induced by ~7-folds in the scenario of pre-treatment of KXS_2012_ plus NGF (1.5 ng/mL; Figure 5B). Similarly, the Erk1/2 phosphorylation (~42/44 kDa) was also being markedly up-regulated with pre-treatment of KXS_2012_, while no significant difference, compared to NGF group, was observed in the co-treatment (Figure 5C).

**Figure 6:**
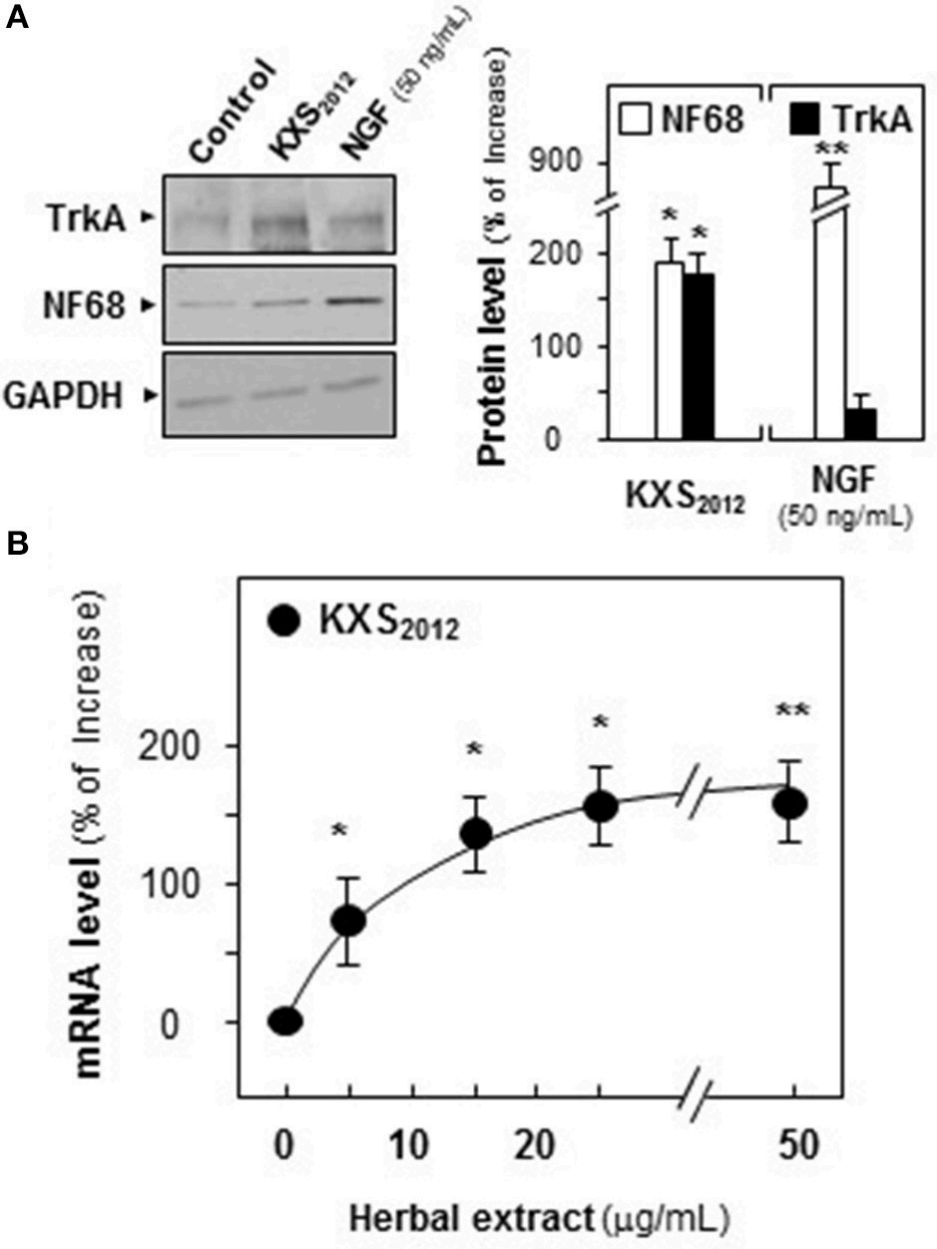
KXS_2012_ increases the expression of TrkA. **(A)** Cultured PC12 cells were treated with KXS_2012_ (15 μg/mL) for 48 h. The cell lysates were collected to determine the expression of Trk A. GAPDH served as a loading control. Quantification plot was shown in right panel. **(B)** Cultured PC12 cells were treated with KXS_2012_ (5–25 μg/mL) for 24 h. The cell lysates were collected to determine the mRNA level of Trk A. Quantification plot was shown. Values are expressed as percentage of increase, where control value is set as 0, Mean ± SEM, *n* = 4. ^*^p < 0.05 and ^**^p < 0.01 compared to the control.

To explore the role of KXS_2012_ in NGF signaling, the expression of Trk A in cultured PC12 cells were analyzed. The expression of Trk A at ~140 kDa, recognized by its specific antibody, was increased by 160% in KXS_2012_ (15 μg/mL)-treated cultures (Figure 6A). NF68 served as a positive control. The mRNA encoding Trk A was analyzed by real-time PCR. Application of KXS_2012_ in PC12 cultures induced Trk A mRNA in a dose-dependent manner: the maximal induction of over 150% increase was revealed at ~25 μg/mL (Figure 6B). This was in consistent with the phosphorylation study of KXS_2012_ that the up-regulation of Trk A expression could be an outcome of increasing sensitivity to low level of NGF during differentiation in PC12 cells.

**Figure 7:**
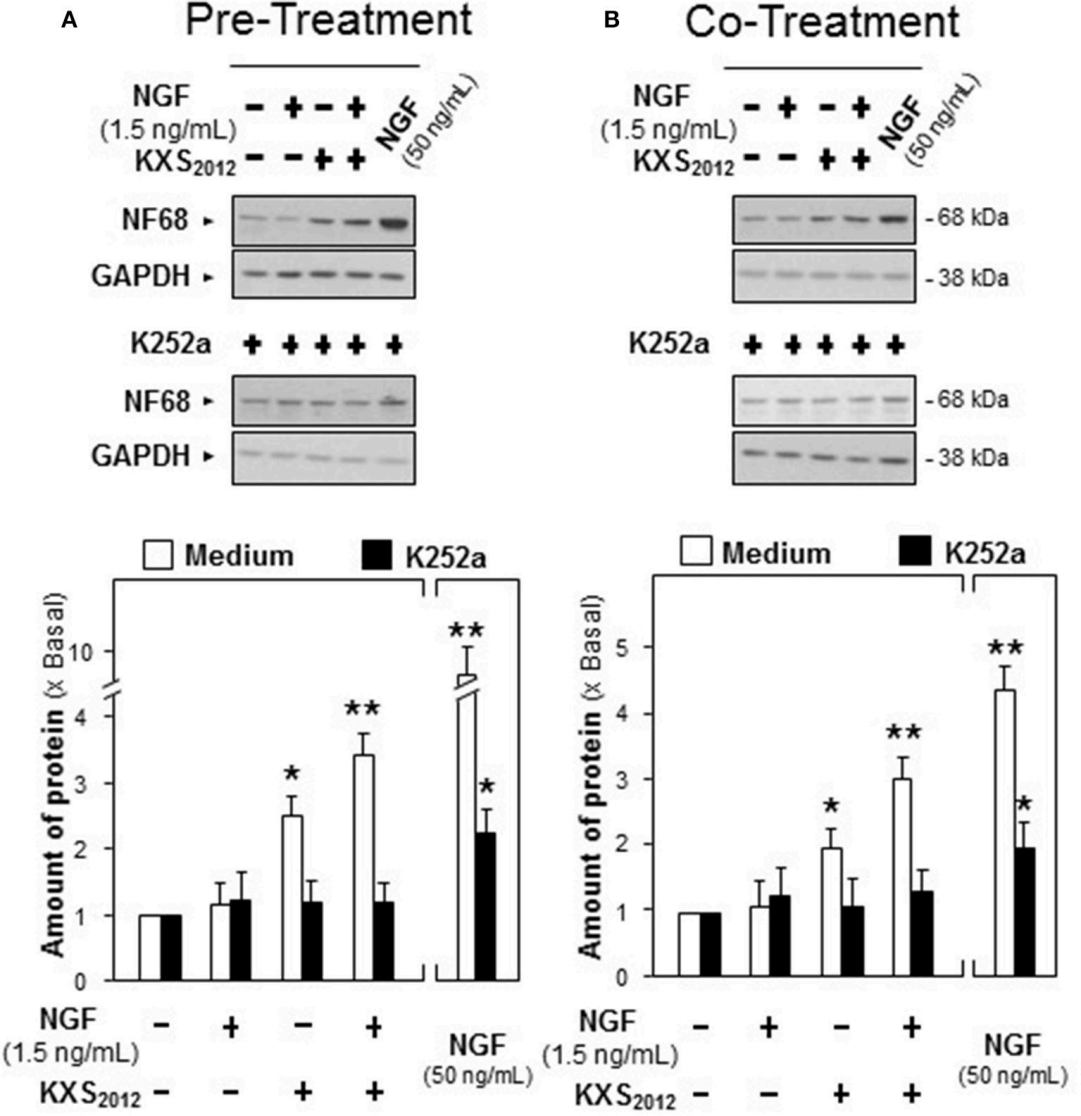
K252a inhibits KXS_2012_ -promoted neurofilament expression. **(A)** K252a (0.1 μM) was applied onto cultured PC12 cells, 3 h before KXS_2012_ (15 μg/mL) treatment. After 48 h, PC12 cells were washed by PBS twice and treated with NGF (1.5 ng/mL) for 24 h. **(B)** K252a (0.1 μM) was applied onto cultured PC12 cells 3 h before KXS_2012_ (15 μg/mL) treatment together with NGF (1.5 ng/mL) for 24 h. The cell lysates were collected to determine the expression of NF68. GAPDH served as a loading control. Quantification plot was shown in low panel. Values are expressed as × Basal, where control value is set as 1, Mean ± SEM, *n* = 4. ^*^p < 0.05 and ^**^p < 0.01 compared to the control.

Moreover, K252a, an inhibitor of tyrosine phosphorylation of Trk A, was applied onto cultured PC12 cells for 3 h before the pre- and co-treatment procedures. In the presence of K252a (0.1 μM), the protein expression of NF68 was totally decreased to that of control in KXS_2012_-treated cultures (Figure 7), which suggested that KXS_2012_-promoted neuronal differentiation was mediated by Trk A. These findings suggested that KXS_2012_ promoted neuronal differentiation by up-regulating Trk A expression, which could increase the cellular response to low level of NGF.

## Discussion and conclusion

Depression is a common mental disorder, which shows severe neuron loss and dysfunction, mood disorder and behavioral disturbance (Barlow and Durand, 2011; Masi and Brovedani, 2011). In addition to neurodegenerative symptoms, depression is more likely to progress to suicide and other concurrent metabolic disorders, e.g., diabetes and cardiovascular diseases (Compare et al., 2014; Koponen et al., 2015). However, current anti-depressants are not able to deal with all the depression-related injuries, which make efficient drug development for anti-depression a must. KXS, a classical Chinese herbal formula is expected to develop as a new anti-depressant. KXS is composed of two functional paired-herbs, i.e., GR-PR and ATR-PO. According to the theory of syndrome differentiation and treatment variation, the varied combination of paired-herbs in KXS has been recorded in Chinese medical books, which directs possible development of anti-depressants (Sun, 1997; Yasunori, 2011). Thus, a re-formulated KXS_2012_ was prepared by optimizing the pairing of GR-PR and ATR-PO: the new formula presented robust anti-depressant action in animal study. It could also induce the expressions of neurotrophic factors as well as their receptors in rat cortical and hippocampal neurons (Yan et al., 2016). However, the underlying mechanism of KXS_2012_ against depression has not been fully investigated. In the study, we investigated the cellular mechanism of KXS_2012_ against depression by using PC12 cell as an *in vitro* model.

It is suggested that depression is largely caused by an impairment of neurogenesis in the brain. During neurogenesis, neuronal differentiation, in presence of neurite outgrowth, plays a critical role in synapse formation, which predicts a promising target of anti-depression (Hattiangady and Shetty, 2010; Snyder et al., 2011). Here, the potential drug targets and cellular mechanism on neuronal differentiation were addressed. The cultured PC12 cells are probably the most valid cell model for the detection of neuronal differentiation. The status of differentiated PC12 cells was determined morphologically through measuring neurite outgrowth and biochemically through analyzing the expression of neurofilaments (Schimmelpfeng et al., 2004; Xu et al., 2012). Our results indicated that KXS_2012_ significantly potentiated NGF-induced neuronal differentiation. The potentiating effect was mediated through NGF receptor, Trk A. KXS_2012_ greatly up-regulated Trk A signaling in the cultures, and the potentiating effect was blocked by k252a, an inhibitor of Trk A. Interestingly, the promoting mechanism of KXS_2012_ in Trk A phosphorylation did not show great effect on the co-treatment situation. This discrepancy could be accounted by KXS_2012_-induced Trk A expression in cultured PC12 cells, which required longer time for Trk A activation. Moreover, the phosphorylations of Erk 1/2 and CREB, the downstream activators of Trk A, were explored here. The co-treatment of KXS_2012_ and NGF did not show marked induction on Erk 1/2 and CREB phosphorylations, as compared to NGF group, which was in accord with our hypothesis that the promoting effect of KXS_2012_ might require longer time of activation. By up-regulating Trk A expression and activation, KXS_2012_ could increase the cellular response to low level of NGF, as to improve neuron adaptation and depression (Welberg, 2008; Peña et al., 2014). In line to this hypothesis, the study on siRNA knockdown of Trk A expression in cultured PC12 cells will be carried out as to further illustrate the function of KXS_2012_ via Trk A signaling.

Considering the major ingredients in KXS_2012_ responsible for anti-depression, different chemicals abundant in the herbal extract have been studied. GR-derived ginsenosides were reported to attenuate depression-like symptom by balancing neuronal cell proliferation and apoptosis and up-regulating neurotransmitter system (Xue et al., 2006; Li et al., 2014; Zhang et al., 2017). PR-derived 3,6′-disinapoyl sucrose reversed mental disorder by improving hippocampal neuron plasticity and neurotrophic signaling pathway in stressed rats (Hu et al., 2010). Asarones, derived from ATR, were more likely to pass the blood brain barrier, which may help improve distributions of other ingredients in the brain (Lu et al., 2014). The finding was consistent with our preliminary results that GR-derived ginsenoside Rb_1_ and Rg_1_, PR-derived 3,6′-disinapoyl sucrose, ATR-derived α-asarone and β-asarone were able to promote neuronal differentiation in neuronal cells. Therefore, it is reasonable to use KXS_2012_ as a new regimen for anti-depression for its robust effect on neuronal differentiation and Trk A signaling.

## Author contributions

LY and KT: conceived and designed the experiments; LY: performed the experiments; LY, MW, SX, and PS: analyzed the data; CB, JL, AX, and TD: contributed reagents; LY, AG, and KT: contributed to the writing of the manuscript.

### Conflict of interest statement

The authors declare that the research was conducted in the absence of any commercial or financial relationships that could be construed as a potential conflict of interest.

## Supplementary material

Supplementary Figure 1 NGF induces neurite outgrowth. Cultured PC12 cells were treated with NGF (0.5–50 ng/mL) for 24 h **(A)** or 48 h **(B)**. Cells were fixed with ice-cold 4% paraformaldehyde. Bar = 10 μm. The % of differentiated cell and length of neurite were counted as described in the Materials and Methods section. Values are expressed as % of total cells in 100 counted cells, Mean ± SEM, *n* = 4. ^*^*p* < 0.05 and ^**^*p* < 0.01 compared to the control.

Supplementary Figure 2 NGF induces the expressions of neurofilaments. Cultured PC12 cells were treated with NGF (0.5–50 ng/mL) for 24 h **(A)** or 48 h **(B)**. The cell lysates were collected to determine the expressions of NF68 (~68 kDa), NF160 (~160 kDa), and NF200 (~200 kDa). GAPDH (~38 kDa) served as a loading control (upper panel). Quantification plot was shown in lower panel. Values are expressed as × Basal, where control value is set as 1, Mean ± SEM, *n* = 4. ^*^*p* < 0.05 and ^**^*p* < 0.01 compared to the control.

Supplementary Figure 3 NGF increases Trk A phosphorylation in a dose-dependent manner. Cultured PC12 cells, serum starvation for 5 h, were treated with NGF (0.5–15 ng/mL) for 10 min. Total Trk A and phosphorylated Trk A were revealed by using specific antibodies. *n* = 4.

## References

[B1] Barlow D. H. Durand V. M. (2011). Abnormal Psychology: An Integrated Approach. Cambridge: Wadsworth Publishing.

[B2] Compare A. Zarbo C. Shonin E. Van Gordon W. Marconi C. (2014). Emotional regulation and depression: a potential mediator between heart and mind. Cardiovasc. Psychiatry Neurol. 2014:324374. 10.1155/2014/324374 25050177 PMC4090567

[B3] Dang H. Sun L. Liu X. Peng B. Wang Q. Jia W.. (2009). Preventive action of Kai Xin San aqueous extract on depressive-like symptoms and cognition deficit induced by chronic mild stress. Exp. Biol. Med. 234, 785–793. 10.3181/0812-RM-354 19429857

[B4] Duman R. S. Aghajanian G. K. (2015). Synaptic dysfunction in depression: potential therapeutic targets. Science 338, 68–72. 10.1126/science.1222939 23042884 PMC4424898

[B5] Ferrari F. Villa R. F. (2016). The neurobiology of depression: an integrated overview from biological theories to climnical evidence. Mol. Neurobiol. 54, 4847–4865. 10.1007/s12035-016-0032-y 27510505

[B6] Hattiangady B. Shetty A. K. (2010). Decreased neuronal differentiation of newly generated cells underlies reduced hippocampal neurogenesis in chronic temporal lobe epilepsy. Hippocampus 20, 97–112. 10.1002/hipo.20594 19309040 PMC2801779

[B7] Hu Y. Liao H. B. Dai H. G. Liu P. Wang Y. Y. Rahman K. (2010). Antidepressant-like effects of 3,6′-disinapoyl sucrose on hippocampal neuronal plasticity and neurotrophic signal pathway in chronically mild stressed rats. Neurochem. Int. 56, 461–465. 10.1016/j.neuint.2009.12.004 20018220

[B8] Hu Y. Zhou X. J. Liu P. Dong X. Z. Mu L. H. Chen Y. B. (2014). Antidepressant and neuroprotective effect of the Chinese herb kaixinsan against lentiviralsh RNA knockdown brain-derived neurotrophic factor-induced injury *in vitro* and *in vivo*. Neuropsychobiology 69, 129–139. 10.1159/000358089 24776773

[B9] Koponen H. Kautiainen H. Leppänen E. Mäntyselkä P. Vanhala M. (2015). Association between suicidal behaviour and impaired glucose metabolism in depressive disorders. BMC Psychiatry 15:163. 10.1186/s12888-015-0567-x 26199013 PMC4509469

[B10] Krishnan V. Nestler E. J. (2008). The molecular neurobiology of depression. Nature 455, 894–902. 10.1038/nature07455 18923511 PMC2721780

[B11] Li J. Du J. Liu D. Cheng B. Fang F. Weng L.. (2014). Ginsenoside Rh1 potentiates dexamethasone's anti-inflammatory effects for chronic inflammatory disease by reversing dexamethasone-induced resistance. Arthritis Res. Ther. 16:R106. 10.1186/ar4556 24887434 PMC4060561

[B12] Lortie K. Huang D. Chakravarthy B. Comas T. Hou S. T. Lin-Chao S.. (2005). The gas7 protein potentiates NGF-mediated differentiation of PC12 cells. Brain Res. 1036, 27–34. 10.1016/j.brainres.2004.12.024 15725398

[B13] Lu J. Fu T. Qian Y. Zhang Q. Zhu H. Pan L.. (2014). Distribution of α-asarone in brain following three different routes of administration in rats. Eur. J. Pharm. Sci. 63, 63–70. 10.1016/j.ejps.2014.06.006 25008114

[B14] Marín-Vicente C. Guerrero-Valero M. Nielsen M. L. Savitski M. M. Gómez-Fernández J. C. Zubarev R. A.. (2011). ATP enhances neuronal differentiation of PC12 cells by activating PKCα interactions with cytoskeletal proteins. J. Proteome Res. 10, 529–540. 10.1021/pr100742r 20973479

[B15] Masi G. Brovedani P. (2011). The hippocampus, neurotrophic factors and depression: possible implications for the pharmacotherapy of depression. CNS Drugs 25, 913–931. 10.2165/11595900-000000000-00000 22054117

[B16] Peña C. J. Bagot R. C. Labonté B. Nestler E. J. (2014). Epigenetic signaling in psychiatric disorders. J. Mol. Biol. 426, 3389–3412. 10.1016/j.jmb.2014.03.016 24709417 PMC4177298

[B17] Pehrson A. L. Sanchez C. (2014). Serotonergic modulation of glutamate neurotransmission as a strategy for treating depression and cognitive dysfunction. CNS Spectr. 19, 121–133. 10.1017/S1092852913000540 23903233 PMC3968911

[B18] Qin Y. J. Zeng Y. S. Zhou C. C. Li Y. Zhong Z. Q. (2008). Effects of Rhodiola rosea on level of 5-hydroxytryptamine, cell proliferation and differentiation, and number of neuron in cerebral hippocampus of rats with depression induced by chronic mild stress. Zhongguo Zhong Yao Za Zhi 33, 2842–2846. 19260327

[B19] Schimmelpfeng J. Weibezahn K. F. Dertinger H. (2004). Quantification of NGF-dependent neuronal differentiation of PC-12 cells by means of neurofilament-L mRNA expression and neuronal outgrowth. J. Neurosci. Methods 139, 299–306. 10.1016/j.jneumeth.2004.05.010 15488244

[B20] Snyder J. S. Soumier A. Brewer M. Pickel J. Cameron H. A. (2011). Adult hippocampal neurogenesis buffers stress responses and depressive behaviour. Nature 476, 458–461. 10.1038/nature10287 21814201 PMC3162077

[B21] Sun S. M. (1997). Beiji Qianjin Yaofang. Beijing: People's Medical Publishing House.

[B22] Vaudry D. Stork P. J. Lazarovici P. Eiden L. E. (2002). Signaling pathways for PC12 cell differentiation: making the right connections. Science 296, 1648–1649. 10.1126/science.1071552 12040181

[B23] Welberg L. (2008). Molecular neuroscience: stress hormones Trk neurons into survival. Nat. Rev. Neurosci. 9, 328–329. 10.1038/nrn2382

[B24] Xiong A. Yan A. L. Bi C. W. Lam K. Y. Chan G. K. Lau K. K.. (2016). Clivorine, an otonecine pyrrolizidine alkaloid from Ligularia species, impairs neuronal differentiation via NGF-induced signaling pathway in cultured PC12 cells. Phytomedicine 23, 931–938. 10.1016/j.phymed.2016.06.006 27387401

[B25] Xu S. L. Choi R. C. Zhu K. Y. Leung K. W. Guo A. J. Bi D.. (2012). Isohamnetin, a flavonol aglycone from Ginkgo biloba L., induces neuronal differentiation of cultured PC12 cells: potentiating the effect of nerve growth factor. Evid. Based Complement. Alternat. Med. 2012:278273. 10.1155/2012/278273 22761636 PMC3385709

[B26] Xue J. F. Liu Z. J. Hu J. F. Chen H. Zhang J. T. Chen N. H. (2006). Ginsenoside Rb1 promotes neurotransmitter release by modulating phosphorylation of synapsins through a cAMP-dependent protein kinase pathway. Brain Res. 1106, 91–98. 10.1016/j.brainres.2006.05.106 16836988

[B27] Yan L. Hu Q. Mak M. S. Lou J. Xu S. L. Bi C. W.. (2016). A Chinese herbal decoction, reformulated from Kai-Xin-San, relieves the depression-like symptoms in stressed rats and induces neurogenesis in cultured neurons. Sci. Rep. 6:30014. 10.1038/srep30014 27444820 PMC4957105

[B28] Yan L. Xu S. L. Zhu K. Y. Lam K. Y. Xin G. Maiwulanjiang M.. (2015). Optimizing the compatibility of paired-herbs in an ancient Chinese herbal decoction Kai-Xin-San in activating neurofilament expression in cultured PC12 cells. J. Ethnopharmacol. 162, 155–162. 10.1016/j.jep.2014.12.049 25560671

[B29] Yasunori N. (2011). Yi Xin Fang. Beijing: Huaxia Publishing House.

[B30] Zhang H. Zhou Z. Chen Z. Zhong Z. Li Z. (2017). Ginsenoside Rg3 exerts anti-depressive effect on an NMDA-treated cell model and a chronic mild stress animal model. J. Pharmacol. Sci. 134, 45–54. 10.1016/j.jphs.2017.03.007 28461003

[B31] Zhang Y. Moheban D. B. Conway B. R. Bhattacharyya A. Segal R. A. (2000). Cell surface Trk receptors mediate NGF-induced survival while internalized receptors regulate NGF-induced differentiation. J. Neurosci. 20, 5671–5678. 10908605 10.1523/JNEUROSCI.20-15-05671.2000 PMC6772538

[B32] Zhu K. Y. Fu Q. Xie H. Q. Xu S. L. Cheung A. W. Zheng K. Y.. (2010). Quality assessment of a formulated Chinese herbal decoction, Kaixinsan, by using rapid resolution liquid chromatography coupled with mass spectrometry: a chemical evaluation of different historical formulae. J. Sep. Sci. 33, 3666–3674. 10.1002/jssc.201000498 21077129

[B33] Zhu K. Y. Mao Q. Q. Ip S. P. Choi R. C. Dong T. T. Lau D. T.. (2012). A standardized Chinese herbal decoction, kai-xin-san, restores decreased levels of neurotransmitters and neurotrophic factors in the brain of chronic stress-induced depressive rats. Evid. Based Complement. Alternat. Med. 2012:149256. 10.1155/2012/149256 22973399 PMC3437946

[B34] Zhu K. Y. Xu S. L. Choi R. C. Yan A. L. Dong T. T. Tsim K. W. (2013). Kai-xin-san, a Chinese herbal decoction containing Ginseng Radix et Rhizoma, Polygalae Radix, Acori Tatarinowii Rhizoma, and Poria, stimulates the expression and secretion of neurotrophic factors in cultured astrocytes. Evid. Based Complement. Alternat. Med. 2013:731385. 10.1155/2013/731385 24222781 PMC3814066

