# Supplementary figures and images for: A Modified Chinese Herbal Decoction (Kai-Xin-San) Promotes NGF-Induced Neuronal Differentiation in PC12 Cells via Up-Regulating Trk A Signaling
Source: Front Cell Dev Biol. 2017 Dec 22;5:118. doi: 10.3389/fcell.2017.00118 (PMC5744097; doi:10.3389/fcell.2017.00118)

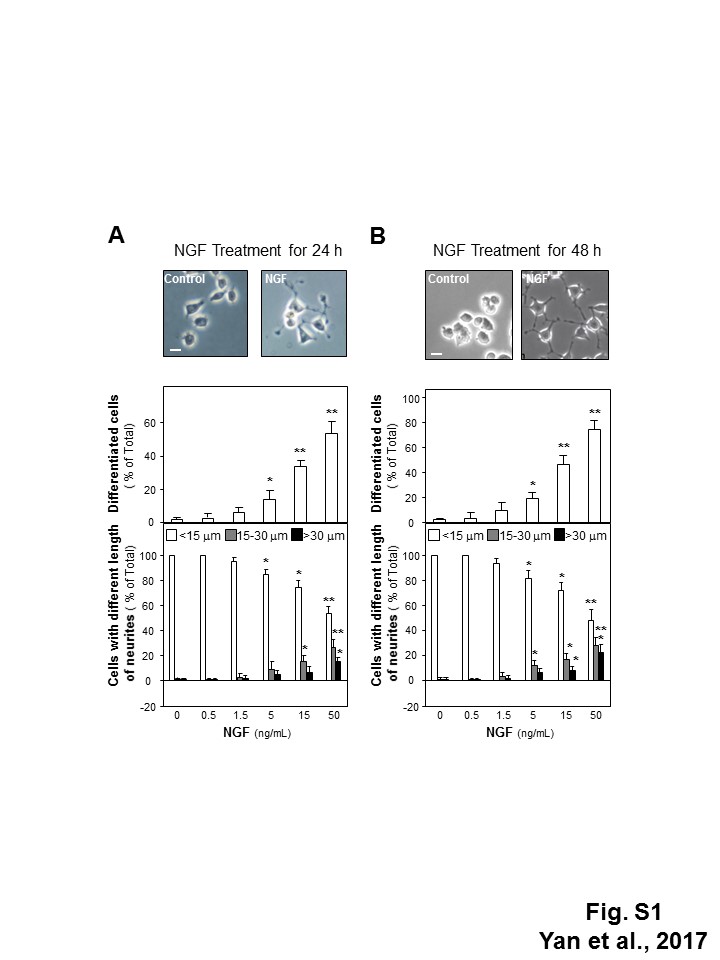

Supplement: Supplementary Figure 1 — NGF induces neurite outgrowth. Cultured PC12 cells were treated with NGF (0.5–50 ng/mL) for 24 h (A) or 48 h (B). Cells were fixed with ice-cold 4% paraformaldehyde. Bar = 10 μm. The % of differentiated cell and length of neurite were counted as described in the Materials and Methods section. Values are expressed as % of total cells in 100 counted cells, Mean ± SEM, n = 4. *p < 0.05 and **p < 0.01 compared to the control. [file Image1.JPEG]

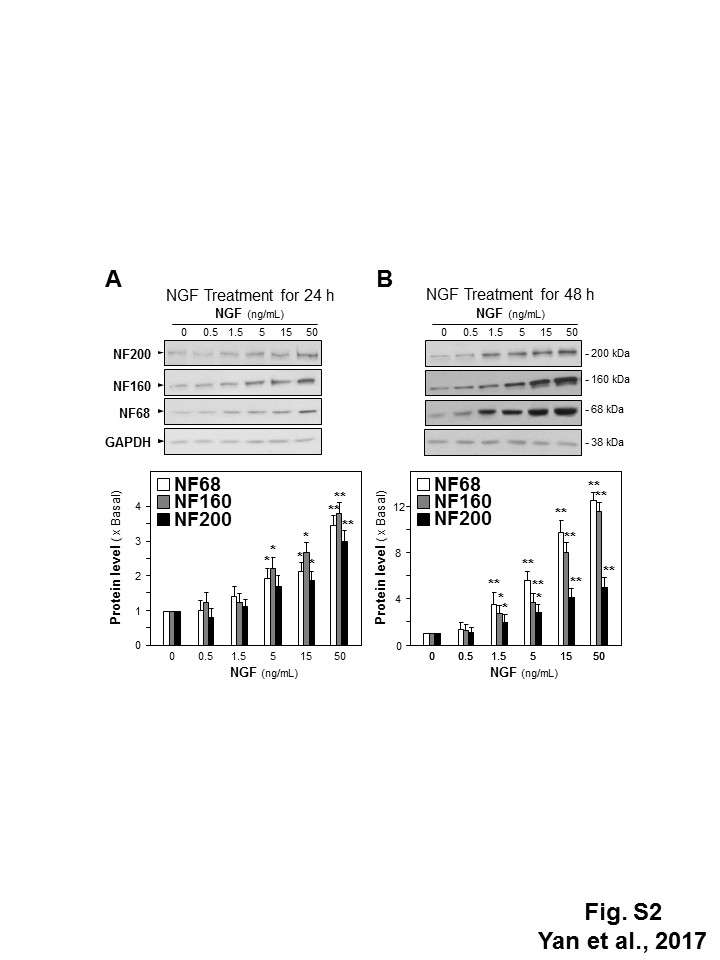

Supplement: Supplementary Figure 2 — NGF induces the expressions of neurofilaments. Cultured PC12 cells were treated with NGF (0.5–50 ng/mL) for 24 h (A) or 48 h (B). The cell lysates were collected to determine the expressions of NF68 (~68 kDa), NF160 (~160 kDa), and NF200 (~200 kDa). GAPDH (~38 kDa) served as a loading control (upper panel). Quantification plot was shown in lower panel. Values are expressed as × Basal, where control value is set as 1, Mean ± SEM, n = 4. *p < 0.05 and **p < 0.01 compared to the control. [file Image2.JPEG]

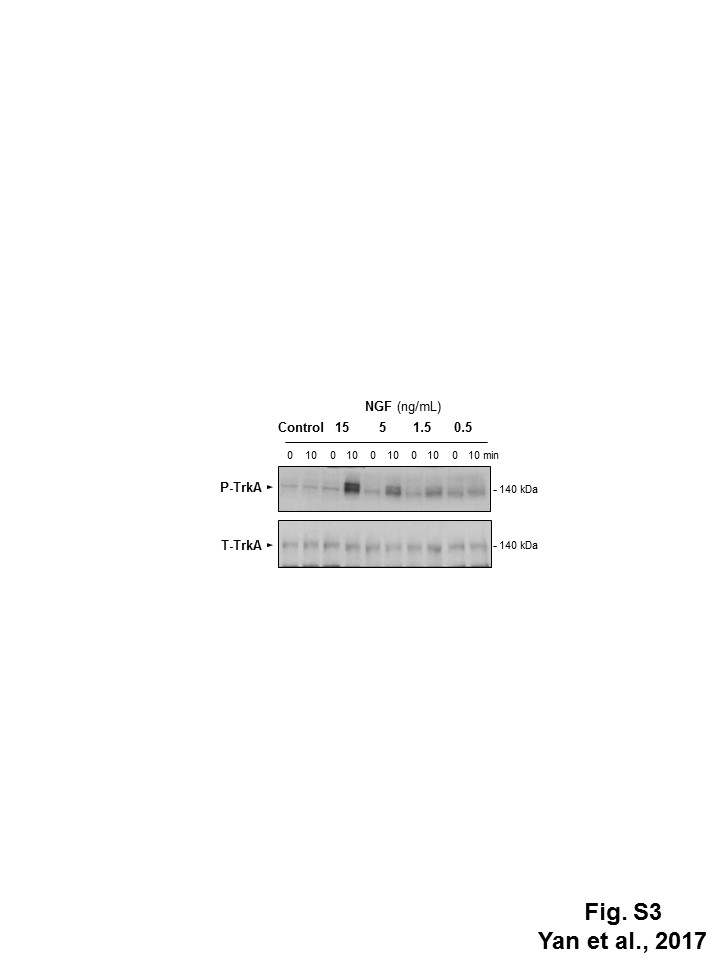

Supplement: Supplementary Figure 3 — NGF increases Trk A phosphorylation in a dose-dependent manner. Cultured PC12 cells, serum starvation for 5 h, were treated with NGF (0.5–15 ng/mL) for 10 min. Total Trk A and phosphorylated Trk A were revealed by using specific antibodies. n = 4. [file Image3.JPEG]
